# Plasma and cerebrospinal fluid interleukin-1β during lipopolysaccharide-induced systemic inflammation in ewes implanted or not with slow-release melatonin

**DOI:** 10.1186/s40104-017-0206-0

**Published:** 2017-10-01

**Authors:** Janina Skipor, Marta Kowalewska, Aleksandra Szczepkowska, Anna Majewska, Tomasz Misztal, Marek Jalynski, Andrzej P. Herman, Katarzyna Zabek

**Affiliations:** 1 Institute of Animal Reproduction and Food Research, Polish Academy of Sciences, Olsztyn, Poland; 2 The Kielanowski Institute of Animal Physiology and Nutrition, Polish Academy of Sciences, Jablonna n/Warsaw, Olsztyn, Poland; 3 Veterinary Medicine Faculty, University of Warmia and Mazury, Olsztyn, Poland; 4 Department of Sheep and Goat Breeding, Animal Bioengineering Faculty, University of Warmia and Mazury in Olsztyn, Olsztyn, Poland

**Keywords:** Albumin, Cerebrospinal fluid, Ewes, Interleukin −1β, LPS, Melatonin

## Abstract

**Background:**

Interleukin-1β (IL-1β) is important mediator of inflammatory-induced suppression of reproductive axis at the hypothalamic level. At the beginning of inflammation, the main source of cytokines in the cerebrospinal fluid (CSF) is peripheral circulation, while over time, cytokines produced in the brain are more important. Melatonin has been shown to decrease pro-inflammatory cytokines concentration in the brain. In ewes, melatonin is used to advance the onset of a breading season. Little is known about CSF concentration of IL-1β in ewes and its correlation with plasma during inflammation as well as melatonin action on the concentration of IL-1β in blood plasma and the CSF, and brain barriers permeability in early stage of lipopolysaccharide (LPS)-induced inflammation.

**Methods:**

Systemic inflammation was induced through LPS administration in melatonin- and sham-implanted ewes. Blood and CSF samples were collected before and after LPS administration and IL-1β and albumin concentration were measured. To assess the functions of brain barriers albumin quotient (QAlb) was used. Expression of IL-1β (*Il1B)* and its receptor type I (*Il1r1*) and type II (*Il1r2*) and matrix metalloproteinase (*Mmp*) 3 and 9 was evaluated in the choroid plexus (CP).

**Results:**

Before LPS administration, IL-1β was on the level of 62.0 ± 29.7 pg/mL and 66.4 ± 32.1 pg/mL in plasma and 26.2 ± 5.4 pg/mL and 21.3 ± 8.7 pg/mL in the CSF in sham- and melatonin-implanted group, respectively. Following LPS it increased to 159.3 ± 53.1 pg/mL and 197.8 ± 42.8 pg/mL in plasma and 129.8 ± 54.2 pg/mL and 139.6 ± 51.5 pg/mL in the CSF. No correlations was found between plasma and CSF IL-1β concentration after LPS in both groups. The QAlb calculated before LPS and 6 h after was similar in all groups. Melatonin did not affected mRNA expression of *Il1B*, *Il1r1* and *Il1r2* in the CP. The mRNA expression of *Mmp3* and *Mmp9* was not detected.

**Conclusions:**

The lack of correlation between plasma and CSF IL-1β concentration indicates that at the beginning of inflammation the local synthesis of IL-1β in the CP is an important source of IL-1β in the CSF. Melatonin from slow-release implants does not affect IL-1β concentration in plasma and CSF in early stage of systemic inflammation.

## Background

It is well established that immune stress caused by infections and inflammatory diseases reduces animals productivity and is a powerful modulator of mechanisms regulating reproduction at all levels of the hypothalamic–pituitary–gonadal axis [1]. A few pathways have been suggested to be responsible for the immune-mediated inhibition of reproductive activity at the level of hypothalamus and one of these involves pro-inflammatory mediators such as cytokines [2]. Hypothalamic interleukin (IL)-1β and tumor necrosis factor (TNF)-α mediates the lipopolysaccharide (LPS)-induced suppression of gonadotropin releasing hormone (GnRH) and luteinizing hormone (LH) release in female rats [3]. In ewes, the central IL-1β is an important modulator of the GnRH biosynthesis and release during immune/inflammatory challenge. The thesis about the crucial role of these cytokines in the transmission of signals from the immune to neuroendocrine systems seems to be supported by the presence of IL-1β and its receptors in the hypothalamus in LPS-treated ewes [4].

In general, passage of molecules from the periphery to the brain is restricted by brain barriers: blood-brain barrier (BBB) located in cerebromicrovascular endothelial cells and blood-cerebrospinal fluid barrier (BCSFB) located in the epithelial cells of choroid plexus (CP) [5]. The origin of central pro-inflammatory cytokines is differentiated. At the beginning of inflammation, the main source of cytokines present in the cerebrospinal fluid (CSF) is peripheral circulation, while over time of inflammation, endogenous cytokines produced in the brain seems to be more important. In rats treated intravenously with IL-1β two waves of cellular activation at the brain appears, the first one at the blood side of the BBB 30 min after IL-1β administration and then after 3 h at the parenchymal side of the BBB [6]. It has been demonstrated in rats, that early after (5 h) injection of IL-1β to the brain BBB becomes permeable to intravenously administered contrast [7]. In this process matrix metalloproteinases (MMPs) as enzymes that catalyze the proteolytic cleavage of basal lamina components and thus remodeling of the extracellular matrix and brain barriers permeability play an important role [8]. Interestingly, melatonin attenuated BBB hyperpermeability in IL-1β stimulated rat brain microvessels endothelial cells in vitro as well as in vivo in mouse traumatic brain injury model [9]. From the periphery, IL-1β is transported throughout the brain barriers [10]. Transport of IL-1β has been suggested to occur via a type II IL-1 receptor [11]. This receptor may also be released from cells and function as decoy receptor to block IL-1β action in contrast to type I IL-1β receptor that transduce IL-1 signals after binding with IL-1β [12, 13]. Expression of type II IL-1 receptor mRNA (*Il1r2*) and I IL-1β receptor (*Il1r1*) was detected in the brain endothelial cells [14] and in the CP [15]. In ewes, intravenous injection of LPS is one model of systemic inflammation which has been used to study mechanisms responsible for the immune-mediated inhibition of reproductive activity [4]. So far little is known about CSF concentration of IL-1β in ewes and its correlation with plasma concentration during LPS-induced systemic inflammation. Melatonin receptors MT1 and MT2 has been demonstrated in the ovine CP what unable direct melatonin action on the CP [16]. Melatonin action on the concentration of IL-1β in blood plasma and the CSF is particularly interesting due to the use of melatonin from continuous slow-release implants to advance the onset of a breeding season in sheep and goats [17].

The present study aimed at evaluating effect of LPS alone and with melatonin slow-release implants on the concentration of IL-1β in blood plasma and the CSF as well as on the BBB permeability in ewes early after LPS administration. Additionally, we evaluated effect of melatonin implantation on mRNA expression of *Il1B* and its receptors *Il1r1 and Il1r2* as well as *Mmp3* and *Mmp9* in the CP under the influence of IL-1β.

## Methods

### Animals and experimental design

**Fig. 1:**
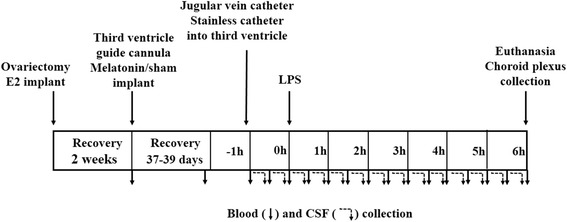
Schematic diagram of the experimental design. At the beginning of May all the ewes (*n* = 14) were ovariectomized and implanted with an oestradiol (E2). Two weeks later, animals were implanted under general anaesthesia with stainless steel guide cannulae into the third ventricle and ewes were melatonin- (*n* = 7) or sham-implanted (*n* = 7). Approximately 40 d later, melatonin- and sham-implanted ewes were treated with lipopolysaccharide (LPS). Blood samples were collected for melatonin, cortisol albumin and IL-1β concentrations measurement. Cerebrospinal fluid (CSF) samples were collected for albumin and IL-1β concentrations measurement. The choroid plexuses were collected 6 h after LPS administration

All animal procedures were conducted in accordance with the Polish Guide for the Care and Use of Animals and approved by the Local Ethics Committee (No. 25/2012). Female adult sheep (4–5 years old, 50–60 kg body weight) of the Blackheaded Mutton breed (*n* = 14) were maintained indoors under natural lighting conditions (latitude 52°N, 21°E) and fed a constant diet of hay, straw and commercial concentrates according to the recommendations proposed by the National Research Institute of Animal Production for adult ewes. Water and mineral licks were available ad libitum. On the beginning of May, ewes were ovariectomized under general anaesthesia and then (middle of May) subcutaneously implanted with an oestradiol (E2) implant, which maintained plasma E2 concentrations of 2–4 pg/L [18]. After 2 wk of recovery ewes were implanted under general anaesthesia with stainless steel guide cannulae (1.2 mm o.d.) into the third ventricle as described earlier [19]. In the middle of May, ewes were first sampled for blood and then one- half of the animals was randomly melatonin-implanted (*n* = 7, slow-release implant of 18 mg, Melovine Ceva Sante Animale, France) and the second half of the animals was sham-implanted (*n* = 7). Approximately 40 d later, ewes were implanted with the jugular vein catheter early on the morning (7:00 am) and have been placed in individual cages where they could lie down and have access to hay and water. To prevent the stress of social isolation, all ewes had visual contact. After that stainless catheter was introduced into the third ventricle and control blood and CSF samples were collected. Then immune stress was induced by intravenously injection of LPS from *Escherichia coli* 055:B5 (Sigma, USA), at the dose of 400 ng/kg of body weight, dissolved in saline (0.9% *w*/*v* NaCl) at a concentration of 10 mg/L (10 μg/mL) as it was used previously in ewes [20]. The individual body mass of experimental ewes were at the range of 52 kg to 63 kg, therefore injection volume of LPS solution/saline was at the range of 2.1 to 2.5 mL. Body temperature was measured before and after LPS administration. Blood plasma was collected just before and after melatonin implantation and then every hour after LPS administration (Fig. 1). Blood samples were collected through a catheter inserted into the jugular vein. First 2 mL of blood samples were removed then 10 mL were collected into the tubes with stabilizer (EDTA). Immediately after centrifuging, the blood plasma were divided into separate (1 mL) aliquots and stored at −40 °C until assayed for IL-1β and albumin. To collect the CSF samples, a stainless steel catheter (1.0 mm o.d., 0.8 mm i.d.) was carefully introduced into the guide cannula and connected to a special cannula-Eppendorf tube system joined to a PHD 2000 infuse/withdrawal pump (Hugo Sachs Elektronik Harvard Apparatus, Germany). The CSF collection from the third ventricle of conscious ewes was performed during a 7 h period (1 h before and 6 h after LPS administration) at a rate of 20 μL/min. The tubes of CSF samples were kept in an ice bath during sampling, and the volume of one sample collected during the 30 min period was about 500 μL. Immediately after filling, the tubes were stored at −80 °C until assayed for IL-1β and albumin. The ewes were euthanized 6 h after LPS administration. After decapitation, the brains were dissected, CP were removed from their anchoring to the Galien’s vein and the split was made along the mid-line, separating the CP from each lateral ventricle. CPs were then immediately frozen in liquid nitrogen and stored at −80 °C until use.

### Hormones and IL-1β concentration measurement

The ability of the melatonin implants to maintain permanently high blood concentrations of melatonin was monitored by determining melatonin concentrations in blood plasma samples obtained before and 40 d after melatonin-implantation using radioimmunoassay (RIA) described by Misztal et al. [21]. The assay sensitivity for melatonin was 16.8 ± 8.0 pg/mL, and the intra- and interassay coefficients of variations were 10.5 and 13.2%, respectively. Blood plasma and CSF IL-1β concentrations were determined by commercially available enzyme-linked immunosorbent assay (ELISA Kit for ovine Interleukin 1β, Cloud-Clone Corp., USA), following the manufacturer’s instructions. The optical density of individual wells was measured by a spectrophotometric microplate reader (Epoch, BioTek, Switzerland) at a wavelength of 450 ± 10 nm. The concentration of IL-1β in samples were determined by comparing the optical density of the samples to the standard curve. The detection limit of the assay was less than 5.9 pg/mL.

### Western blotting and CSF/blood plasma quotient

To assess the functions of BCSFB albumin quotient (QAlb) was used. The CSF and blood plasma samples (equal volume 16 μL CSF and diluted blood plasma (1:160) with addition of 4 μL of loading buffer) from the sham- and melatonin-implanted group (one animal from each group) were loaded onto 10% sodium dodecyl sulfate (SDS) polyacrylamide gels together with serial dilutions of sheep serum albumin (SSA, Biorbyt, USA, range 0.25 to 5 μg/μL). Electrophoresis was performed using the MiniProtean II electrophoretic apparatus (BioRad, USA) at 60 mV constant voltage. Thereafter, proteins were transferred onto 0.2 μm thick nitrocellulose membranes (Whatman Inc., Germany) at 30 V for 1.5 h in a semi-dry transfer system (BioRad, USA). After 1.5 h blocking with block buffer (TBST, 50 g/L nonfat milk in 10 mL Tris buffer saline containing 0.5% Tween 20) at room temperature, the membranes were extensively washed in TBST and incubated overnight at 4 °C with rabbit polyclonal antibodies against sheep albumin at 1:300 dilution (Anitbodies-online, Germany). After final wash, membranes were developed using chemiluminescence SuperSignal® West Dura Kit (Thermo Scientific, USA) and visualized by VersaDoc 4000 MP Imaging System (BioRad, USA). Based on a SSA dilution curve, the albumin concentration was calculated in both CSF and blood plasma samples by measuring optical densities of the bands (Image Lab 5.2.1, Software, BioRad, USA). The integrity of BCSFB was estimated by the ratio of albumin concentrations in CSF and blood plasma. The albumin quotient was evaluate as follows: QAlb = Alb (CSF)/ Alb (blood plasma).

### Relative gene expression assays

The total RNA from the CP was isolated using NucleoSpin RNA II Kit (Marcherey-Nagel, Germany). All steps of the isolation were performed according to the manufacturer’s protocol. The purity and concentration of the isolated RNA were quantified spectrophotometrically using a NanoDrop 1000 instrument (Thermo Fisher Scientific, USA). The integrity of RNA was verified by electrophoresis using 1.2% agarose gel stained with ethidium bromide (Sigma Aldrich, USA). To synthesize cDNA, the DyNAmo cDNA Synthesis Kit (Thermo Fisher Scientific, USA) and 1 μg of total RNA were used. Expression of interleukin 1β (*Il1B)* and its receptor type I (*Il1r1*) and type II (*Il1r2*) and matrix metalloproteinases (*Mmp*) 3 and 9 in the ovine CP was determined by real-time PCR. Specific primer pairs for the different genes were used according to the literature or were designed using Primer-BLAST (National Center for Biotechnology Information) and were synthesized by Genomed (Poland) and are presented in Table 1. One reaction mixture for real-time PCR reaction (10 μL) contained 3 μL of diluted (1:14 reference genes, 1:10 *Il1B*, *Il1r1*, *Il1r2* and 1:8 *Mmp3* and *Mmp9*) cDNA, 0.2 μmol/L of the forward and reverse primers and 5 μL of mastermix from a DyNAmo SYBR Green qPCR Kit with ROX (Thermo Fisher Scientific, USA). The following protocol was used: 95 °C for 10 min for Hot Start modified Tbr DNA polymerase, followed by 35 cycles of 15 s of denaturation at 95 °C, 30 s of annealing at X °C (see Table 1) and 30 s of extension at 72 °C. After the cycles, a final melting curve analysis under continuous fluorescence measurement was performed to evaluate the specific amplification. The results were analyzed using Real-time PCR Miner (on-line available: http://www.miner.ewindup.info/version2), based on the algorithm developed by Zhao and Fernald [22].

**Table 1:** Sequences of oligonucleotide primers used for real time-PCR

GenBank Acc. No.	Gene	Amplicon size, bp	Temp. of primers annealing, °C	Forward/ reverse	Sequence 5′ → 3′	Reference
X54796.1	*Il1B*	137	59	forward	CAGCCGTGCAGTCAGTAAAA	[35]
				reverse	GAAGCTCATGCAGAACACCA	
NM_001206735.1	*Il1r1*	124	59	forward	GGGAAGGGTCCACCTGTAAC	[35]
				reverse	ACAATGCTTTCCCCAACGTA	
NM_001046210	*Il1r2*	161	59	forward	CGCCAGGCATACTCAGAAA	[36]
				reverse	GAGAACGTGGCAGCTTCTTT	
XM_004015970.1	*Mmp3*	112	60	forward	AAGGCAGACATTTTTGGCGG	Originally designed
				reverse	ATGCCTCTTGGGGAACCTGC	
XM_004014614.1	*Mmp9*	115	60	forward	CTTCCGATGGAAAGAACGGGC	Originally designed
				reverse	GGGATCACAACGCCTTTGC	
NM_001034034	*Gapdh*	143	60	forward	TGACCCCTTCATTGACCTTC	[27]
				reverse	GATCTCGCTCCTGGAAGATG	
NM_001009784.1	*Actb*	122	60	forward	GCCAACCGTGAGAAGATGAC	[27]
				reverse	TCCATCACGATGCCAGTG	
BC_108088.1	*Hdac1*	115	60	forward	CTGGGGACCTACGGGATATT	[35]
				reverse	GACATGACCGGCTTGAAAAT	

### Statistical analysis

All data are presented as the mean ± standard error of the mean [SEM]. The real-time PCR results are presented as the relative gene expression of the target gene vs. the mean of 3 reference genes (*Gapdh*, *Actb*, *Hdac1*). The body temperature, melatonin and IL-1β concentrations, were analyzed by a one-way ANOVA for repeated measures and relative gene expression by t-test using PRISM 6 GraphPad Software (San Diego, USA). For statistical analysis, percentage data of QAlb were multiplied by 0.1 and then arcsin transformed according to the eq. (Y = deg.(arcsin(sqrt(Y/100))) using PRISM 6 GraphPad Software. The transformed data were subjected to one-way ANOVA. Additionally, the differences between QAlb in sham-and melatonin-implanted ewes before LPS administration were analyzed by Welch test. The relationship between variables was analyzed using Pearson’s correlation coefficient. Statistical significance was assumed at *P* < 0.05.

## Results

**Fig. 2:**
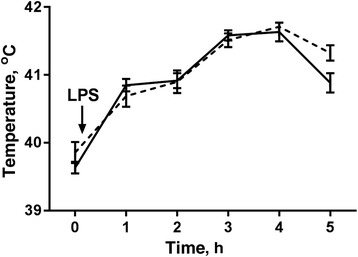
The mean (±SEM) body temperature before and every 1 h for 5 h after LPS treatment in sham-implanted (solid line) and melatonin-implanted (dotted line) adult ewes

The mean body temperature before the LPS administration was 39.6 ± 0.1 °C in the sham-implanted group and 39.9 ± 0.2 °C in the melatonin-implanted group and increased significantly (*P* ≤ 0.05) to 41.6 ± 0.1 °C and 41.7 ± 0.1 °C 4 h after LPS administration, respectively (Fig. 2). Additionally, in all ewes LPS administration induced rapid breathing and shortness of breath, sneezing, and stopped feed intake, anhedonia and reduced social interactions which are collectively termed ‘sickness behavior’.The plasma melatonin concentration in melatonin-implanted ewes increased significantly (*P* < 0.05) from 8.2 ± 2.2 pg/mL (before implantation) to 84.6 ± 15.0 pg/mL (one mo after implantation) while in sham-implanted stayed on the level of 9.6 ± 1.1 pg/mL. As shown on Table 2 immunoreactive IL-1β was not detected in blood plasma collected 1 h before LPS in 2 out of 6 ewes in sham-implanted group and in 4 out of 7 ewes in melatonin-implanted group. In other ewes IL-1β concentration ranged from 49.4 to 197.2 pg/mL and 128.6 to 192.7 pg/mL in sham- and melatonin-implanted ewes, respectively. Two and a half h after LPS administration the concentration of IL-1β increased in all investigated animals and reached the mean level of 159.3 ± 53.1 pg/mL and 197.8 ± 42.8 pg/mL in sham- and melatonin-implanted ewes, respectively. Melatonin did not affect IL-1β concentration in blood plasma. In CSF collected before LPS, IL-1β was not detected in 2 out of 7 ewes in melatonin group. In sham-implanted ewes IL-1β ranged from 13.5 to 51.7 pg/mL while in other melatonin-implanted ewes ranged from 11.8 to 67.4 pg/mL. After LPS treatment the mean concentration of IL-1β increased in all investigated animals in sham-implanted (129.8 ± 54.2 pg/mL) and melatonin-implanted (139.6 ± 51.5 pg/mL) ewes. There was no effect (*P* > 0.05) of melatonin on CSF IL-1β concentration as well as no differences (*P* > 0.05) between IL-1β in blood plasma and CSF after LPS administration in both groups. No correlations was found between plasma and CSF IL-1β concentration after LPS administration in both sham- and melatonin-implanted groups (r^2^ = 0.08; *P* < 0.29 vs. r^2^ = 0.01; *P* < 0.4).

**Table 2:** Individual measurements of IL-1β concentration (pg/mL) in blood plasma and cerebrospinal fluid (CSF) in sham-implanted and melatonin implanted ewes before and 3 h after lipopolysaccharide (LPS, 400 ng/kg) administration

Sham-implanted					Melatonin-implanted				
	Plasma, pg/mL		CSF, pg/mL			Plasma, pg/mL		CSF, pg/mL	
Ewe #	Before	After LPS	Before	After LPS	Ewe #	Before	After LPS	Before	After LPS
1	0.0	65.3	27.8	48.0	1	143.3	211.0	26.4	410.8
2	0.0	105.9	13.5	35.5	2	128.	134.7	67.4	146.5
3	53.0	58.5	21.0	50.4	3	0.0	210.7	19,2	39.6
4	76.1	113.0	20.3	365.6	4	0.0	298.4	0.0	198.5
5	197.2	399.0	22.8	66.7	5	0.0	80.3	0.0	12.3
6	49.4	214.2	51.7	213.7	6	192.7	378.7	24.2	54.0
					7	0.0	71.0	11,8	115.4
Mean (±SEM)	62.0 (±29.7)	159.3^a^ (±53.1)	26.2 (±5.4)	129.8^a^ (±54.2)		66.4 (±32.1)	197.8^a^ (±42.8)	21.3 (±8.7)	139.6^a^ (±51.5)

^a^significant vs. concentration before LPS administration at *P* < 0.05

**Fig. 3:**
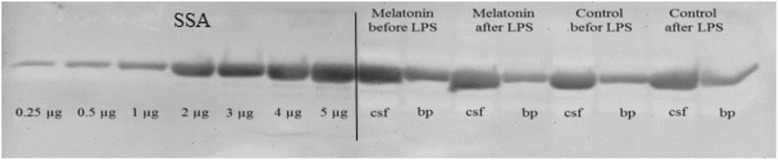
Determination of albumin levels by western blot analysis in sheep cerebrospinal fluid (csf) and blood plasma samples (bp) collected before and 6 h after LPS administration. The densities of the bands were based on sheep serum albumin (SSA) dilution curve

**Fig. 4:**
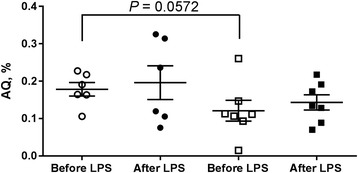
Mean (±SEM) cerebrospinal fluid albumin quotient (QAlb) before and after lipopolysaccharide (LPS, 400 ng/kg) administration in sham- (circles) and melatonin-implanted (squares) adult ewes

**Fig. 5:**
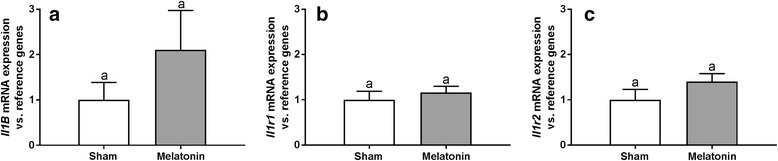
The effect of sham- (white) and melatonin-implantation (grey) on the mean (±SEM) relative relative gene expression for interleukin 1β (*Il1B*; **a**) and its receptor type I (*Il1r1*; **b**) and type II (*Il1r2*; **c**) in the choroid plexus of lipopolysaccharide (LPS)-treated adult ewes. Data presented on each panel were normalized to the average relative quantity of target gene expression in sham- and melatonin-implanted, which was set to 1.0

The albumin concentrations in the CSF collected from the third brain ventricle and blood plasma were calculated on the base of linear dilution curve of sheep serum albumin detected by western blot method (Fig. 3) and then the integrity of BCSFB was estimated by ratio of albumin concentrations in the CSF and blood plasma (QAlb). Six hours after LPS administration mean QAlb was on the level of 0.20% ± 0.05 and 0.14% ± 0.02 (*P* > 0.05) in sham- and melatonin-implanted ewes and was similar to that observed before LPS administration (0.18% ± 0.02 vs. 0.12% ± 0.03, *P* = 0.0572, Fig. 4). mRNA expression of *Il1B* and its receptors *Il1r1* and *Il1r2* in the CP collected 6 h after LPS administration were similar (*P* > 0.05) in both sham- and melatonin-implanted group (Fig. 5). Within 35 amplification cycles mRNA expression of *Mmp3* and *Mmp9* was not detected.

## Discussion

Our results show that in ovariectomized and E2 treated ewes LPS in a dose of 400 ng/mL increased IL-1β concentration in blood plasma and the CSF early (2.5 h) after LPS administration. In our previous study LH secretion as well as the GnRH release was observed to be suppressed 3 h after intravenous LPS administration in ewes [23]. The peripheral administration of LPS increases pro-inflammatory cytokine level IL-1β, TNFα and IL-6 in blood plasma in many animals, however, there are species differences in a dose of LPS necessary to trigger the response [24–26]. Ewes are much more sensitive to LPS in comparison to mice and rats in which the range of the applied LPS doses is from 5 μg/kg to 5 mg/kg. In our study the mean concentration of IL-1β reach the level of 159.3 ± 53.1 pg/mL in plasma and 129.8 ± 54.2 pg/mL in CSF in control and 197.8 ± 42.8 pg/mL in plasma and 139.6 ± 51.5 pg/mL in CSF of melatonin-implanted ewes. Individual plasma IL-1β levels were differentiated in both groups but means were similar to these observed in pigs 3 h after continuous LPS infusion in a dose of 250 ng/kg/h [26]. We did not find any significant differences in IL-1β mean concentration between groups (sham- and melatonin-implanted) as well as between compartments (blood plasma and the CSF). This indicates the lack of melatonin slow-release implants effect on IL-1β concentration in both plasma and CSF early (2.5 h) after LPS administration. In our study, daytime plasma melatonin concentration in melatonin-implanted ewes were similar to this observed in our previous study [27] and reported by Skinner and Malpaux [28] for plasma collected at night. We did not observed any correlation between plasma and CSF concentration of IL-1β in both groups, what may suggests that transport of IL-1β from blood to the CSF is not only the source of IL-1β in the CSF early after LPS administration, and that CSF IL-1β originates from other sources. These findings are in line with results described by Qann and colleagues [29], who observed that in rats subseptic doses of LPS (0.01–10 μg/kg) that are in range of the dose used in ewes induced *Il1B* mRNA expression only in the CP, the circumventricular organs and meninges. Moreover, secretion of cytokines to the brain by activated cells of the BBB has been described as an additional, to saturable transport system, pathway of cytokine access to the brain [10].

In addition to evaluating the effect of LPS alone and with melatonin from slow-release implants on the concentration of IL-1β in blood plasma and the CSF, the second aim of this study was to investigate the BBB permeability in ewes early after LPS administration. The integrity of brain barriers was estimated by the ratio of albumin concentrations in CSF and blood plasma. The QAlb, calculated in our study before LPS administration was similar to that obtained by Chen et al. [30] for young (1–2 yr) and middle aged (3–5 yr) ewes. Moreover, the QAlb on the level of 0.18% ± 0.02 in sham-implanted and 0.12% ± 0.03 in melatonin-implanted ewes observed in ewes before LPS administration, despite the difference at the very edge of significance (*P* = 0.0572), seem to confirm previous observation related with melatonin as possible modulator of molecule passage throughout the brain barriers in sheep. These include: 1) higher steroids access to the CSF during long than short days in female sheep [18, 31], 2) higher passage of leptin from the periphery to the CSF in rams during long days than short days [32], 3) higher expression of tight junction proteins in the CP in ewes during short than long days [33] and 4) photoperiod-dependent change in CSF proteome composition in ewes [34]. The lack of differences in QAlb before and after LPS administration, found in our studies, indicates that despite the high level of IL-1β in blood and the CSF the integrity of BBB and BCSFB was not damaged 6 h after LPS administration. Indeed, in the CP collected 6 h after LPS administration the expression of mRNA for *Mmp*3 and 9, that are responsible for degradation of extracellular matrix and therefore for increase of BCSFB permeability was very weak.

## Conclusions

In summary we demonstrated that intravenous LPS administration in ewes induces rapid increase of IL-1β in blood plasma and the CSF that is not modulated by melatonin from slow-release implants. The lack of changes in the brain barrier permeability early after LPS administration, at the time when LPS-dependent suppression of GnRH secretion was observed in ewe, indicates that LPS acts mainly at the BBB and BCSFB which are a place for elaboration of signal molecules that communicate peripheral immune status to the brain.

## Abbreviations

Actb: β-actin
BBB: Blood-brain barrier
BCSFB: Brain-cerebrospinal fluid barrier
bp: Blood plasma
CP: Choroid plexus
CSF: Cerebrospinal fluid
E2: Oestradiol
Gapdh: Glycer-aldehyde-3-phosphate dehydro-genase
GnRH: Gonadotropin releasing hormone
Hdac1: Histone deacetylase 1
IL: Interleukin
IL-1R1: Interleukin 1 receptor type I
IL-1R2: Interleukin 1 receptor type II
LH: Luteinizing hormone
LPS: Lipopolysaccharide
Mmp: Matrix metalloproteinase
MT: Melatonin receptor
QAlb: Albumin quotient
RIA: Radioimmunoassay
SDS: Sodium dodecyl sulfate
SEM: Standard error of the mean
SSA: Sheep serum albumin
TBST: Tris buffer saline with Tween
TNF: Tumor necrosis factor
